# From apparent to true – from frequency to distributions (I)

**DOI:** 10.3325/cmj.2020.61.289

**Published:** 2020-06

**Authors:** Branimir K. Hackenberger

**Affiliations:** Department of Biology, Josip Juraj Strossmayer University, Osijek, Croatia *hackenberger@biologija.unios.hr*

The current COVID-19 pandemic has sparked many questions and re-examinations of the existing epidemiological knowledge. The disease surprised many of those who were trying to make prediction models. Prediction models are extremely important in epidemics since they allow better planning and implementation of epidemiological measures, as well as of activities necessary to reduce the consequences of epidemics. In addition to virulence, ie, the virus's ability to infect and cause disease in the host, several other factors need to be known if we want to monitor the course of the epidemic and the severity of its consequences. One of the most important measures used to describe an epidemic is the prevalence, ie, the proportion of the population that is infected. In practice, a special term, seroprevalence, is used to denote the proportion of the population with antibodies to the pathogen in the serum. In the case of COVID-19, this would be the proportion of the population in which the presence of specific antibodies to the SARS-CoV-2 virus was detected by testing. It is difficult to test the entire population, especially in a short period of time necessary to respond to an epidemic in a timely manner and determine the measures needed to successfully fight it. By testing a part of the population, ie, a sample, the proportion of individuals positive on a pathogen can be calculated – the so-called apparent prevalence. From apparent prevalence, it is necessary to calculate the true prevalence, ie, the prevalence in the entire population. From this, two new problems arise – the sensitivity and specificity of the test. If these two test parameters are equal to 100%, depending on the sample size, ie, the number of tested persons, it is easy to determine the proportion of the entire population that is infected. However, in practice, very often these parameters are less than 100%. Even when it comes to top-notch tests, it can happen that at the time of testing the quantity of virus in a patient is lower than the detection threshold of the test, which will result in a false-negative test. It is also not difficult to imagine a situation in which a false-positive result appears. Test sensitivity is defined as the probability that a positive result will be obtained if the patient is indeed ill. Specificity is also a probability – the probability that the test will give a negative result if the patient is not ill. If either of these two characteristics, ie these two probabilities, is less than 100%, the test is called imperfect. Positive predictive value is the probability that a person with a positive test is indeed ill, and negative predictive value is the probability that a person with a negative test is not ill.

The initial variables for determining the true prevalence are presented in Table 1. According to this table, the sensitivity of the test is Se = a / Nd = p (T + | D +), specificity is Sp = d / Nh = P (T- | D-), positive predictive value PPV = a / Np, and negative predictive value NPV = d / Nn. The accuracy of the test is the probability of a correct test result regardless of whether it is positive or negative, ACC = (a + d) / N. If both the sensitivity and specificity are known, and if the whole sample has been tested with the same test, the true prevalence can be determined by a simple calculation of the Rogan-Gladen estimator (TP) (1).

**Table 1:** Basic variables of testing. “a” is the number of true positives, “b” is the number of false positives, “c” is the number of false negatives, and “d” is the number of true negatives. Np is the number of positive and Nn is the number of negative tests. Nd is the number of persons determined to be ill, and Nh is the number of persons determined to be healthy

		Disease		Sum
+	-
Test	+	True (a)	False (c)	a + c = Np
	-	False (b)	True (d)	b + d = Nn
Sum		a + b = Nd	c + d = Nh	N

TP = (AP + (Sp-1)) / (Sp + (Se-1)) = (AP + (d / Nh) -1))) / ((d / Nh) + ((a / Nd) -1))

The variance of this estimator is equal to Var (TP) = (AP * (1-AP)) / (N (Se + Sp-1) ^ 2). The expression in the denominator (Se + Sp-1) is called the Youden's index (2). This index tells how many times the variance of the true prevalence is greater than the variance of the apparent prevalence. For large samples, and in epidemics there are usually large samples, the confidence interval of the true prevalence can be calculated using the expression:

CI (TP) = ± 1.96 AA ((AP (AP-1)) / (N ((Sp + Se -1) ^ 2)) ^ 0.5

**Figure 1:**
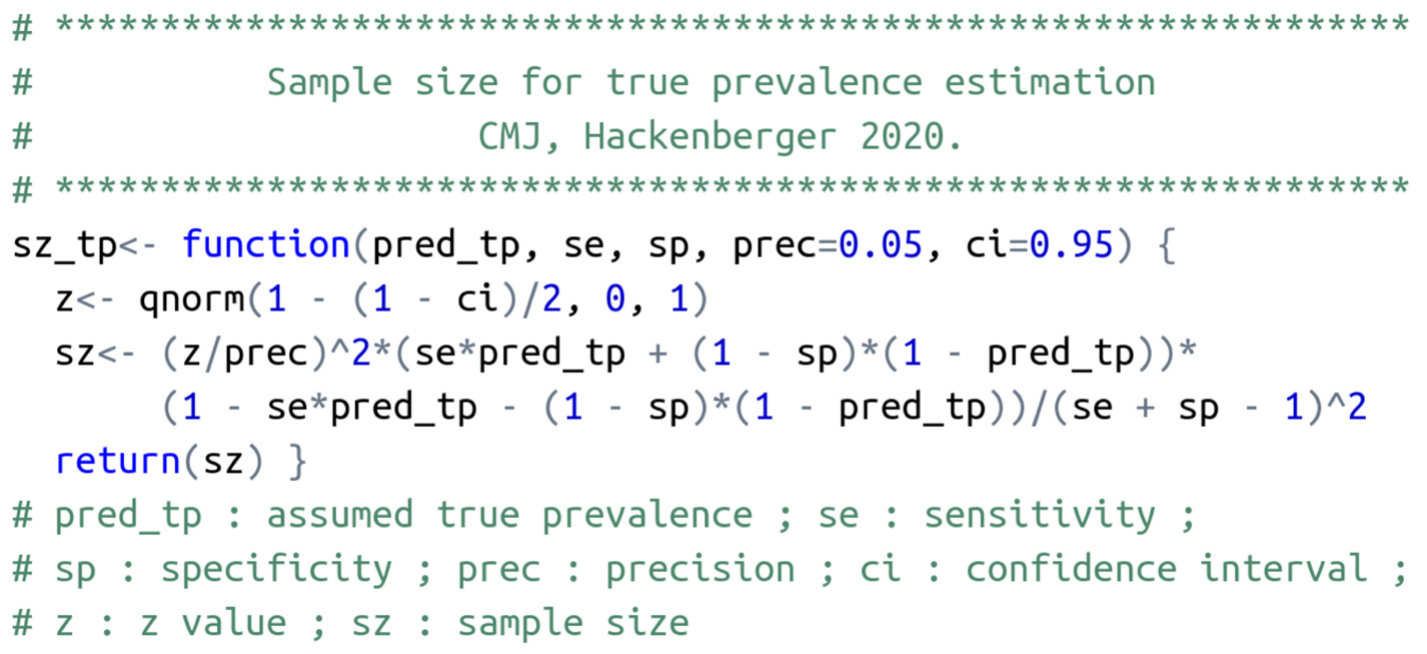
A simple function written in R for the estimation of true prevalence according to sensitivity and specificity of the used test and assumed true prevalence.

One of the most important issues in determining the true prevalence is the issue of sample size. How many people need to be tested if we want to calculate the true prevalence in the population? This question is relatively easy to answer. Namely, the sample size, as always in statistics, depends on several important parameters: the true prevalence, sensitivity and specificity of the test used, and, of course, desired confidence interval. The wider the confidence interval, the smaller the sample we will need. Equally, as test sensitivity and specificity are closer to 1, ie, closer to 100%, we will have to test fewer people to determine the true prevalence within the desired confidence interval. There are several different algorithms for calculating the minimum sample size required for inference with the given parameters. One of the most common is the so-called trial and error procedure. Within the R statistical environment, the sample size required to determine the true prevalence could be very easily determined using the n.tp() function from the *RSurveillance* package. This package has been removed from the CRAN repository and is only available in the repository archives. However, several other packages are available that allow the calculation of the sample size for epidemiological studies, such as *bdpv* (3), *MKmisc* (4), and *epiR* (5). Figure 1 shows the R script of a simple function to calculate the sample size needed for the calculation of the true prevalence if sensitivity and specificity are known and if we assume the true prevalence.

Although everything presented so far is quite trivial, in practice additional problems appear. For example, the COVID-19 pandemic has a special feature. This feature is not uncommon in various diseases, but in COVID-19 it is particularly pronounced. Namely, this disease is especially deadly for certain categories within the population. From the very beginning, it has been obvious that this disease has a lethal effect mainly on the elderly and people with chronic diseases. As it is common that people with health problems seek medical help, it is to be expected that among those tested there will be more people from high-risk subpopulations. Another problem concerning sampling that appeared in the COVID-19 pandemic is that due to the extremely high virulence of the SARS-CoV-2 virus, the risk level of certain groups within the population was very quickly differentiated. Health workers, public service workers, police officers, teachers, counter clerks, etc, are significantly more likely to be infected than other population groups. All the above mentioned true prevalence calculations and the required sample size needed for its calculation refer to a homogeneous randomized sample from the population. The people tested in the countries affected by the pandemic were mostly people who sought medical help or were aware that they had been in contact with an infected person, very often a person from a subset of people with higher risk. Besides, the use of different tests in the same research makes things more complicated. Namely, as already mentioned, various tests and testing methods differ in their sensitivity and specificity. Table 2 shows the declared values of some commercial tests for SARS-CoV-2. If the lowest values for specificity and sensitivity of the testing kits, as well as the highest expected prevalence, are used in calculating the sample size, very often large sample sizes are obtained. One solution to this problem is to conduct a pilot study to investigate the difference in prevalence in more and less vulnerable subpopulations. Such a pilot study might be able to show how the calculated true prevalence in an endangered subpopulation could be transposed to the entire population.

**Table 2:** Sensitivity and specificity of some commercial tests for COVID-19 (SARS-CoV-2 virus) (6)

Type of test	Producer	Declared properties	
sensitivity	specificity
CCI	Diazyme	0.912	0.973
ECLIA	Roche	0.655	0.9981
ELISA	DiaSorin	0.9	0.98
ELISA	Epitope Diagnostics	1	1
ELISA	Creative Diagnostics	1	1
ELISA	Eagle Biosciences	1	1
mod. ELISA	Bio-Rad	0.98	0.99
RDT	Cellex	0.938	0.956
RDT	Autobip Diagnostics	0.957	0.99
RDT	Aytu Biosciences	0.879	1
RDT	sd Biosensor	0.82	0.97
RDT	CTK Biotech	0.969	0.994
RDT	BioMedomics	0.886	0.9063
RDT	RayBiotech	0.841	0.923
RDT	Sure Biotech	0.82	0.94
RDT	Sugentech	0.91	0.967
RDT	Confirm Biosciences	0.931	0.991
RDT	Artron	0.934	0.977
CCI	Diazyme	0.912	0.973
ECLIA	Roche	0.655	0.9981
ELISA	DiaSorin	0.9	0.98

In addition to the usual frequentist approach to calculating true prevalence, Bayesian statistics is increasingly used. Although the Bayesian approach is much more complicated, its main advantage is that it formally includes both preliminary prevalence data and expert data (7).

**Figure 2:**
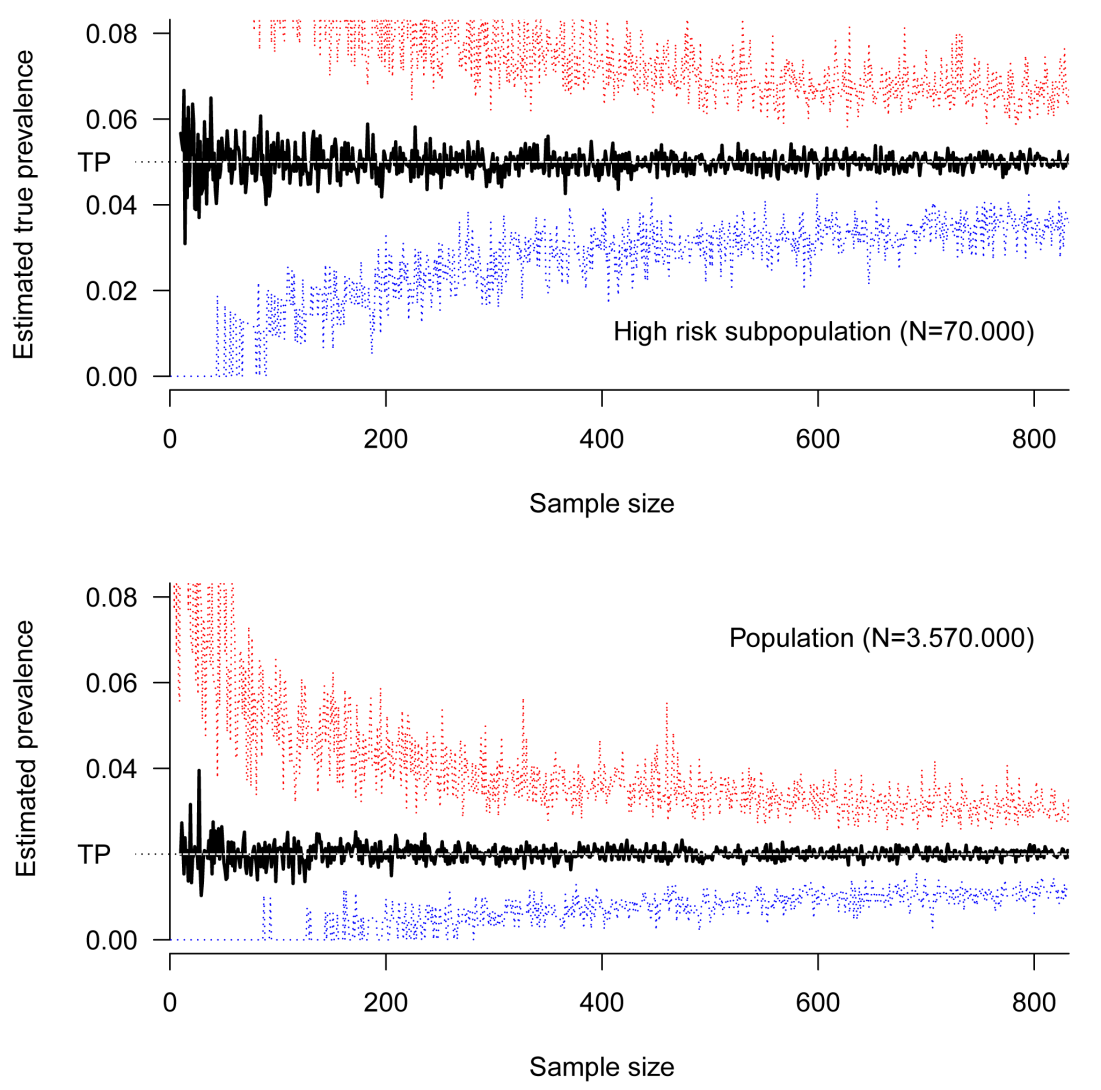
The output of the simulation of infected population sampling. The upper image shows a simulation of sampling of only a high-risk subpopulation and the lower image shows a simulation of sampling of the whole population (including the subpopulation). Red and blue points are extreme values for the estimated true prevalence during the simulation. TP is the true prevalence of virtual populations.

For the COVID-19 pandemic research, our team developed several simulations in which we performed statistical experiments and compared the effectiveness of different statistical methods. Such usage of simulations to perform *in silico* experiments is becoming more common. The main reason for this is the advantage provided by performing *in silico* experiments before and during the final experiment. Namely, if the simulations are made following the existing data and assumptions based on the facts and experience of the research team, they provide both an excellent insight into the possible course of the experiment and the information necessary for optimal design. Figure 2 shows one of the results of such an experiment, which included a virtual population of 3 570 000 people with a subpopulation of 70 000 people whose risk level is 2.5 times higher than the mean risk level in the population. The population was virtually sampled, and the influence of the sample size on the probability of determining the true prevalence as accurately as possible was assessed. A particularly valuable advantage of performing *in silico* experiments with virtual populations is the ability to construct and incorporate rules and knowledge that are gained during a real experiment. Likewise, most good simulations are plastic enough to adapt to the specifics of a particular population (eg, frequency of social interactions, availability of medical care). An interesting comparison of calculating the required sample size by the usual method and calculating it “empirically” using simulation is given in Table 3.

**Table 3:** The sample sizes required to calculate the true prevalence by the usual method and by simulating a virtual population with known true values of the epidemic parameters

Test properties		Supposed true prevalence											
sensitivity	specificity	0.01		0.02		0.03		0.05		0.1		0.2	
			Sim		Sim		Sim		Sim		Sim		Sim
1	1	16	16	31	31	45	45	73	73	139	139	246	246
0.7	0.9	405	423	425	416	445	460	483	489	574	693	733	812
0.9	0.9	232	271	247	285	261	302	290	285	355	451	462	423
0.6	0.9	578	606	602	589	626	728	673	716	784	715	984	944
0.9	0.7	907	1008	917	998	926	1167	944	986	984	1180	1040	1057
0.55	0.55	38,046	44,035	38,061	35,952	38,076	44,696	38,104	48,455	38,169	36,234	38,277	40,279

In the next column, the main features and differences of the frequentist and Bayesian approach to determining the true prevalence and some other epidemiological parameters, as well as the principle of making and using simulations by widely available and free software, will be presented.
